# Electrostatic Secondary‐Sphere Interactions That Facilitate Rapid and Selective Electrocatalytic CO_2_ Reduction in a Fe‐Porphyrin‐Based Metal–Organic Framework

**DOI:** 10.1002/anie.202206085

**Published:** 2022-06-28

**Authors:** Ran Shimoni, Zhuocheng Shi, Shahar Binyamin, Yang Yang, Itamar Liberman, Raya Ifraemov, Subhabrata Mukhopadhyay, Liwu Zhang, Idan Hod

**Affiliations:** ^1^ Department of Chemistry and Ilse Katz Institute for Nanoscale Science and Technology Ben-Gurion University of the Negev Beer-Sheva 8410501 Israel; ^2^ Shanghai Key Laboratory of Atmospheric Particle Pollution and Prevention Department of Environmental Science & Engineering Fudan University Shanghai 200433 China; ^3^ Shanghai Institute of Pollution Control and Ecological Security Department of Environmental Science & Engineering Shanghai 200092 China

**Keywords:** CO_2_ Reduction, Electrocatalysts, Fe-Porphyrin, Metal–Organic Frameworks, Secondary-Sphere

## Abstract

Metal–organic frameworks (MOFs) are promising platforms for heterogeneous tethering of molecular CO_2_ reduction electrocatalysts. Yet, to further understand electrocatalytic MOF systems, one also needs to consider their capability to fine‐tune the immediate chemical environment of the active site, and thus affect its overall catalytic operation. Here, we show that electrostatic secondary‐sphere functionalities enable substantial improvement of CO_2_‐to‐CO conversion activity and selectivity. In situ Raman analysis reveal that immobilization of pendent positively‐charged groups adjacent to MOF‐residing Fe‐porphyrin catalysts, stabilize weakly‐bound CO intermediates, allowing their rapid release as catalytic products. Also, by varying the electrolyte's ionic strength, systematic regulation of electrostatic field magnitude was achieved, resulting in essentially 100 % CO selectivity. Thus, this concept provides a sensitive molecular‐handle that adjust heterogeneous electrocatalysis on demand.

## Graphical abstract

Electrostatic secondary‐sphere interactions provide precise and systematic control over heterogeneous electrocatalytic CO_2_ reduction selectivity and activity in a Fe‐porphyrin based MOF. Specifically, we show that MOFs allow the incorporation of molecular catalysts and positively‐charged ligands one next to the other, in a manner which promotes catalysis by stabilization of reactive intermediates during electrochemical reaction conditions.

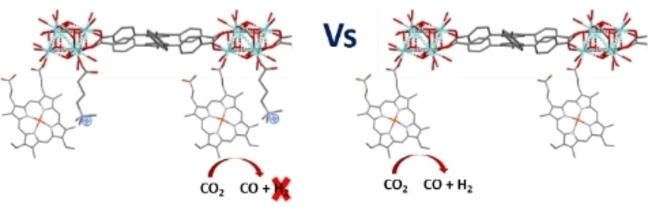

## Introduction

Electrocatalytically driven CO_2_ reduction reaction (CO_2_RR) to produce alternative fuels and chemicals is a useful means to store renewable energy in the form of chemical bonds.^[1]^ CO_2_ is a very stable and relatively inert chemical, and thus its chemical conversion is difficult due to the large amounts of energy needed and effective electrocatalysts required.^[2]^ As a result, in recent years there has been a significant increase in research efforts aiming to develop highly efficient CO_2_RR electrocatalysts.^[3]^ In that respect, several homogeneous molecular catalysts (e.g. FeTPP,^[4]^ CoTPP,^[5]^ Re(bpy)(CO)_3_Cl,^[6]^ Mn(bpy)(CO)_3_Br,^[7]^ and Ni(cyclam)^[8]^) have shown good selectivity and high turnover frequencies toward CO formation. Nevertheless, practical realization of these catalytic materials necessitates their heterogenization into porous, solution‐accessible solid electrodes.

Metal–organic frameworks (MOFs), are a rapidly growing class of crystalline porous coordination polymers, built from periodic networks of multitopic organic ligands and metal ion/cluster containing nodes.^[9]^ The highly modular nature of these unique, high surface area^[10]^ materials have attracted significant interest over the last two decades for various applications such as gas storage^[11]^ and separation,^[12]^ chemical catalysis,^[13]^ artificial photosynthesis,^[14]^ and sensing.^[15]^ Additionally, over the last few years, an emerging subfield aims at the possibility of using MOFs for heterogeneous immobilization of large concentration of molecular catalysts to drive electrochemical catalysis.^[16]^ As an outcome, several proof‐of‐concept reports have shown the capability of MOFs to function as a solid, porous platform for driving electrocatalytic solar fuel generation reactions such as CO_2_ reduction,[^16b^, ^17^] oxygen reduction reaction (ORR),[^16a^, ^18^] oxygen evolution reaction (OER),^[19]^ and hydrogen evolution reaction (HER).^[20]^ These recent studies provide a strong foundation for further research aiming to utilize MOFs for electrocatalytic energy‐conversion schemes. Yet, a further leap in our understanding of electrocatalytic MOF‐based materials must be obtained in order to achieve highly active and selective electrocatalytic systems.

This goal could be realized once other unique MOF assets, e.g. their chemical modularity, well‐defined nature, and structural periodicity, are explored. In fact, judicious use of those properties can serve to systematically modulate the catalytically‐active site's immediate chemical environment. It is well‐known that enzymes which catalyze small molecule activation employ subtle secondary‐sphere interactions to increase catalytic performance.^[21]^ Typically, enzymes use several approaches for secondary‐sphere effects, including pendent proton relays,[^21^, ^22^] bi‐metallic active sites, and charged functional groups. These effects maintain an essential task during operation, as they may stabilize reactive intermediate species and enhance catalysis rate and selectivity. This notion has indeed inspired a significant volume of work, examining the effect of secondary‐sphere interactions on the electrocatalytic activity of homogeneous molecular catalysts.^[23]^ Perhaps the most convincing experimental evidence of the importance of secondary‐sphere moieties in homogeneous CO_2_ electrocatalytic reduction comes from the pioneering work by Savéant.^[24]^ In this study, the substitution of phenol groups (acting as proton relays) for the phenyl groups of the well‐established FeTPP electrocatalyst for the reduction of CO_2_ to CO led to significant increases in activity, demonstrating over 50 million catalytic turnovers and 90 % faradaic efficiency at an overpotential of 0.43 V.

However, examples of incorporation of secondary‐sphere moieties in MOF‐based heterogeneous CO_2_ electroreduction are still rare.^[25]^ Unlike molecular design principles, MOF structures do not require that catalytic and co‐catalytic moieties be covalently linked to achieve cooperative behavior. This introduces a variety of potential catalyst structures which cannot be achieved through small molecule synthesis. Thus, one can envision a design of MOF that incorporate two sets of functionalities: 1) introduction of molecular catalytic elements in spatial registry, and 2) secondary‐sphere functional groups assembled into the MOF structure.

In this work, we demonstrate that the electrochemical CO_2_ reduction rate and selectivity of an Fe‐porphyrin‐based MOF could be systematically tuned via a careful modulation of electrostatic interactions at close proximity to the catalytically active site. As a case study, we have chosen to explore a Fe‐porphyrin (Hemin)‐modified Zr_6_‐oxo based 2D‐MOF, Zr‐BTB (termed Zr‐BTB@Hemin), due to its chemical and structural robustness, and favorable mass‐transport properties. In order to modify the local environment surrounding the catalytic entity, we have post‐synthetically tethered a cationic functional group, (3‐carboxypropyl)trimethylammonium (TMA)^[25a]^ proximal to the Fe‐porphyrin active site. In turn, we found that the installed TMA imposes an electrostatic stabilization of surface‐bound CO intermediate during catalysis, thus greatly enhancing the CO_2_‐to‐CO conversion rate and selectivity. Moreover, through changing TMA's surface density and electrolyte's ionic strength, we were able to vary the extent of electrostatic interactions. By doing so, systematic tailoring of electrocatalytic selectivity was gained, reaching practically up to 100 % CO_2_ conversion to CO.

## Results and Discussion

As a model platform for studying electrocatalytic CO_2_ reduction, we have chosen to use a 2D‐MOF, Zr‐BTB (BTB ‐1,3,5‐Tris(4‐carboxyphenyl) benzene).^[26]^ This layer‐structured MOF possess several important advantages over conventional three‐dimensional MOFs: i) Zr‐BTB's structure contains a 6‐coordinated Zr_6_‐oxo node (or 6 available carboxylate‐anchoring sites), thus allowing high surface loading of immobilized ligands (molecular catalyst or secondary‐sphere groups), ii) It contains a 2D structure which exposes a large portion of solution‐accessible surface and permits better diffusion of reactants towards the catalytic sites.

**Figure 1:**
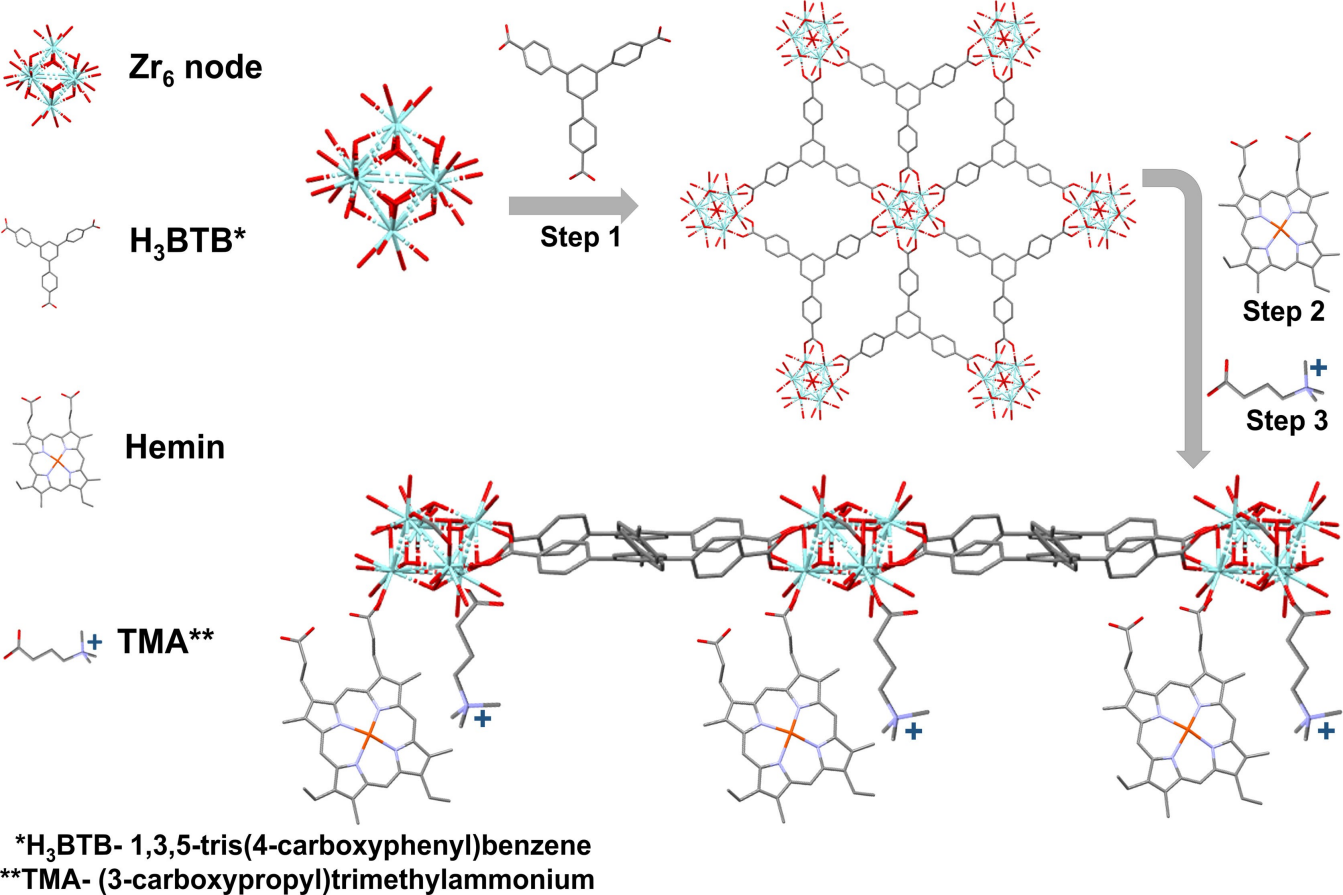
Schematic illustration of Zr‐BTB@Hemin‐TMA synthesis. Step 1, showing the preparation of Zr‐BTB nanosheet. Step 2, post‐synthetic modification with Hemin (molecular catalyst). Step 3, post‐synthetic modification with TMA (electrostatic secondary‐sphere ligand).

To prepare our MOF‐based electrodes, first Zr‐BTB MOF nanosheets were grown using a strategy adapted from a previously reported synthetic procedure,^[27]^ as illustrated in Figure 1, step 1 (for detailed experimental details, see Supporting Information). In short, a 30 ml DMF solution containing 100 mg ZrCl_4_, 100 mg organic ligand 1,3,5‐Tris(4‐carboxyphenyl) benzene (H_3_BTB), 5 ml H_2_O and 6 g of benzoic acid, was put in an 120 °C kept oven for 48 hours. Thereafter, the resulting MOF was thoroughly washed in DMF and then in acetone. Then, the as‐formed Zr‐BTB was post‐synthetically modified with a Hemin CO_2_ reduction molecular catalyst via solvent‐assisted‐ligand‐incorporation (SALI)^[28]^ method (Zr‐BTB@Hemin, Figure 1, step 2). To do so, 100 mg of Zr‐BTB and 160 mg of Hemin were dissolved in 10 ml DMF and kept in an oven at 70 °C overnight, followed by washing with DMF and acetone. Thereafter, the resulting Zr‐BTB@Hemin was deposited on a conductive carbon cloth substrate. A deposition ink was made by mixing 30 mg Zr‐BTB@Hemin in 2 mL of 3 : 1 H_2_O : propanol (v/v) with 70 μL of Nafion binder. An ink volume of 340 μL was then drop‐casted on the carbon cloth, to form a MOF surface loading of 5 mg cm^−2^. In turn, to engender electrostatic secondary‐sphere functionality to the MOF, an additional SALI was performed to tether the positively‐charged TMA ligand on the Zr_6_‐oxo node (Figure 1, step 3), by reacting a Zr‐BTB@Hemin electrode in a 5 ml methanol solution containing 5 mg TMA, at 50 °C overnight (Zr‐BTB@Hemin‐TMA).

**Figure 2:**
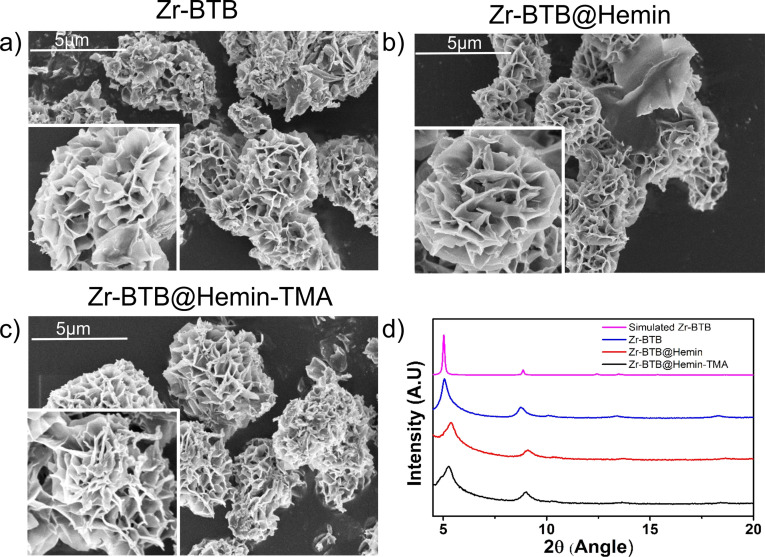
SEM images of a) Zr‐BTB, b) Zr‐BTB@Hemin, and c) Zr‐BTB@Hemin‐TMA. d) PXRD of Zr‐BTB, Zr‐BTB@Hemin, and Zr‐BTB@Hemin‐TMA.

**Figure 3:**
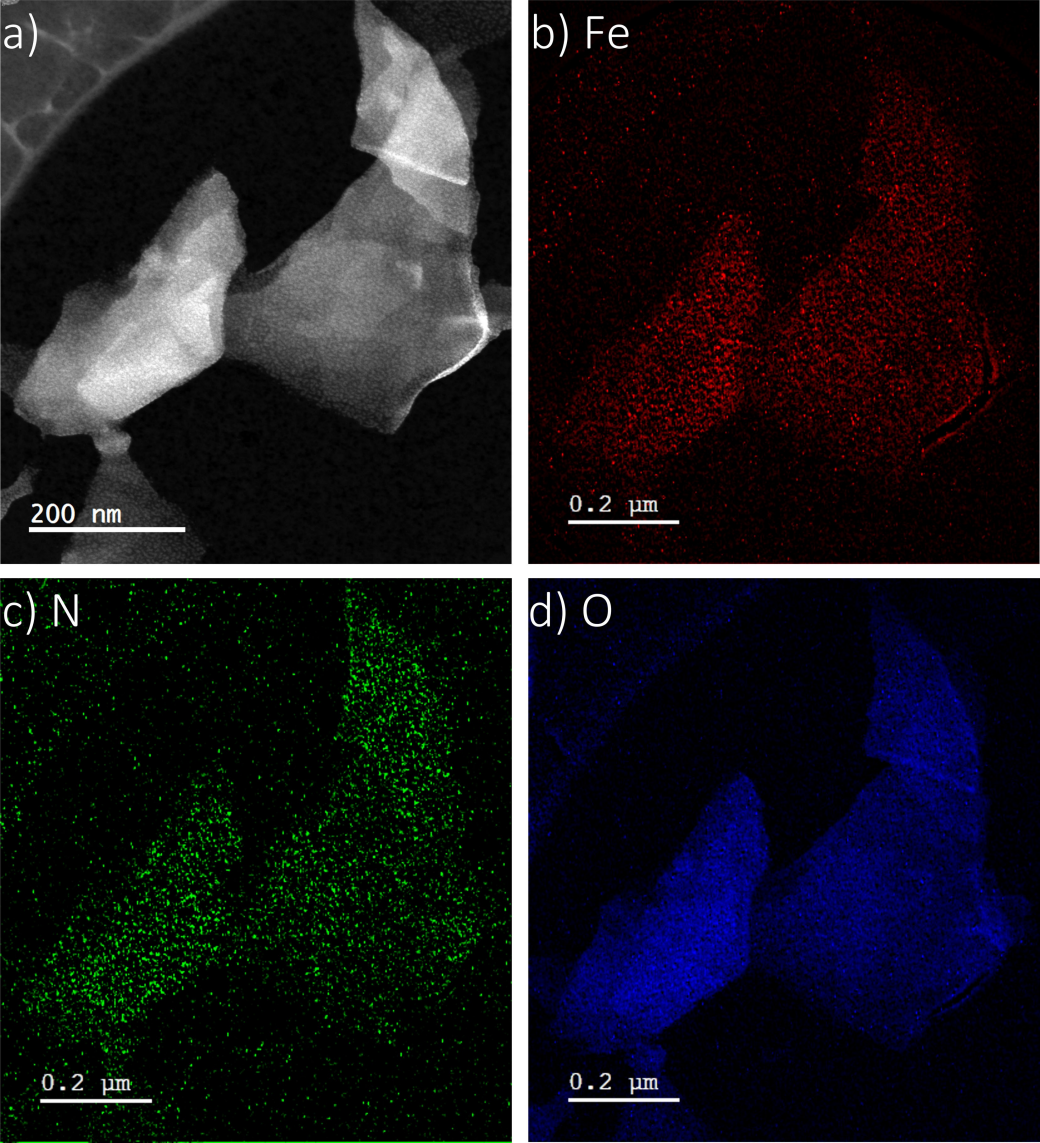
a) HR‐TEM image of Zr‐BTB@Hemin‐TMA. In addition, EELS elemental mapping is included for b) iron, c) nitrogen, and d) oxygen.

**Figure 4:**
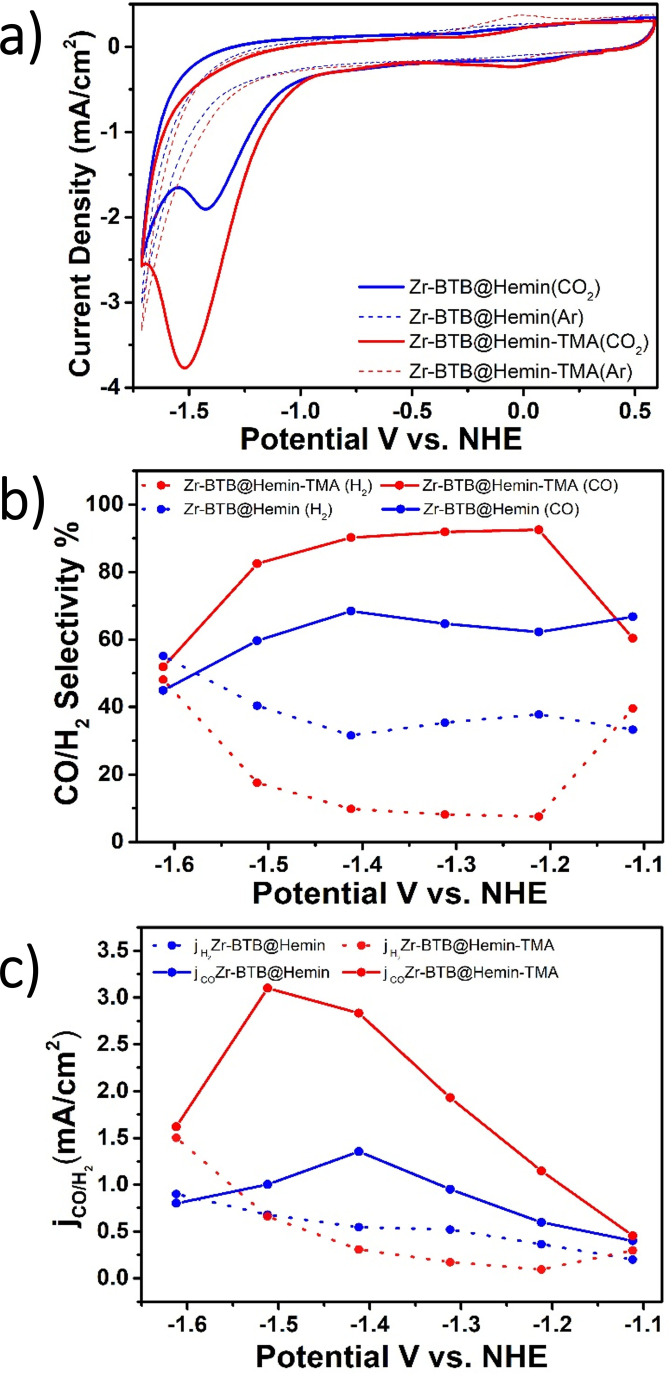
Electrochemical CO_2_ reduction analysis. a) Cyclic voltammetry measurements comparing Zr‐BTB@Hemin (blue), and Zr‐BTB@Hemin‐TMA (red) in both Ar (dashed line) and CO_2_ environment (solid line). b) Comparison of catalytic selectivity towards CO (solid line) and H_2_(dashed line) for Zr‐BTB@Hemin (blue) Zr‐BTB@Hemin‐TMA (red), as a function of applied potential. c) Comparison of partial currents attributed to CO (solid line) and H_2_ (dashed line) for Zr‐BTB@Hemin (blue) Zr‐BTB@Hemin‐TMA (red), as a function of applied potential.

Scanning electron microscopy (SEM) images show that the as‐grown Zr‐BTB MOF exhibits a typical flower‐like 2D nanosheet morphology (Figure 2a). Additionally, as can be seen in Figures 2b and c, modification of the MOF with either the molecular catalyst (Zr‐BTB@Hemin) or TMA (Zr‐BTB@Hemin‐TMA) respectively, did not alter the MOF's nanosheets morphology. Powder X‐ray diffraction (PXRD) analysis conducted for all samples confirm the successful formation of Zr‐BTB MOF,^[29]^ as well as the preservation of its crystal structure integrity upon Hemin and TMA anchoring (Figure 2d). Compared to Zr‐BTB, in Zr‐BTB@Hemin and Zr‐BTB@Hemin‐TMA one can observe a small shift in the PXRD pattern (toward higher angles) which is attributed to a curving of the 2D‐MOF layer, due to an increased surface tension created upon post synthetic ligand modification.^[30]^ Further characterization of the MOF samples was obtained through high‐resolution transmission electron microscopy (HR‐TEM). We were able to exfoliate Zr‐BTB@Hemin‐TMA nanosheets via tip‐ultrasonication and thus record its HR‐TEM image, as shown in Figure 3a. Elemental mapping of Zr‐BTB@Hemin‐TMA (iron, nitrogen, and oxygen) was obtained by electron energy loss spectroscopy (EELS), confirming the incorporation of Hemin (iron) and TMA (nitrogen), as shown in Figures 3b–d respectively. Diffuse reflectance infrared fourier transform spectroscopy (DRIFTS) analysis confirm the covalent anchoring of Hemin and TMA on the MOF's Zr_6_‐oxo node, as evidenced by the diminishing of the terminal OH/OH_2_ IR band upon ligands attachment (Figure S1).[^28b^, ^31^] Additionally, Zr‐BTB@Hemin‐TMA nanosheet thickness was estimated to be ≈4 nm (corresponding to 1–2 layers) using atomic force microscopy (AFM) and EELS analysis (Figure S2). Energy‐dispersive X‐ray spectroscopy (EDS) characterization (point analysis and mapping) was performed, showing the incorporation of Fe (Hemin) in the MOF samples (Figure S3). As shown in Figure S4c, In order to accurately quantify the concentration of MOF‐installed Hemin, inductively coupled plasma ‐ optical emission spectrometry (ICP‐OES) measurements were done, showing Hemin surface loading of 0.88 and 0.87 per Zr_6_‐oxo node for Zr‐BTB@Hemin and Zr‐BTB@Hemin‐TMA respectively. Using a combined ^1^H NMR and ICP‐OES analysis, the loading level of TMA ligand in the MOF was found to be 0.63 per Zr_6_‐oxo node (Figure S4c). We note that similar TMA loading (0.66 per Zr_6_‐oxo node) was achieved when Zr‐BTB@Hemin powder was modified with TMA (compared to Zr‐BTB@Hemin electrode), as seen in Figure 4c. In addition, X‐ray photon electron spectroscopy (XPS) measurement were performed to further characterize Zr‐BTB@Hemin and Zr‐BTB@Hemin‐TMA (Figure S5). Compared to Zr‐BTB@Hemin, Zr‐BTB@Hemin‐TMA exhibits an additional N1s peak located at 402.8 eV, corresponding to the positively charged nitrogen of TMA, thus further confirming its successful incorporation into the MOF.

We then set to investigate the effect of electrostatic secondary‐sphere interactions on the MOF's electrocatalytic CO_2_ reduction properties. Electrochemical analysis was carried out using a gas‐tight H‐cell three‐electrode setup, in CO_2_‐saturated MeCN electrolyte containing 0.1 M LiClO_4_ and 0.1 M trifluoroethanol (TFE) as proton source. Platinum foil, Ag wire, and MOF‐coated carbon cloth were used as counter, quasi‐reference, and working electrodes, respectively. The working and reference electrodes were separated from the counter electrode by an ion‐exchange membrane, Nafion 117 (see Supporting Information for details). Figure 4a presents cyclic voltammetry (CV) measurements comparing Zr‐BTB@Hemin (blue curves) with Zr‐BTB@Hemin‐TMA (red curves) conducted at a scan rate of 50 mV s^−1^, under Ar (dashed curves) and CO_2_ (solid curves) environments. For both samples, in CO_2_ saturated solutions a clear catalytic CO_2_ reduction peak is observed at potentials corresponding to the Hemin's Fe^0/+^ redox couple, thus signaling their electrocatalytic activity.^[4]^ Moreover, it is evident that in the presence of TMA, the catalytic peak current is more than doubled (3.8 mA cm^−2^ compared to 1.8 mA cm^−2^), hence hinting that the electrocatalytic CO_2_ reduction is accelerated by the positively charged groups positioned adjacent to the molecular catalyst.

In order to further evaluate the electrocatalytic activity and selectivity of Zr‐BTB@Hemin and Zr‐BTB@Hemin‐TMA, bulk electrolysis experiments were conducted at an applied potential window of −1.1 V to −1.6 V vs. NHE (Figure S6). As can be seen in Figure 4b, over a wide potential range (−1.2 V to −1.5 V vs. NHE), a drastic enhancement of electrocatalytic CO_2_‐to‐CO selectivity was obtained for Zr‐BTB@Hemin‐TMA electrodes compared to Zr‐BTB@Hemin. Particularly, the highest CO production selectivity, 92 %, was achieved at the potential of −1.2 V vs. NHE, compared to 64 % CO obtained for Zr‐BTB@Hemin (for all bulk electrolysis experiments, the total faradaic efficiency (CO and H_2_) was essentially 100 %, as seen in Figure S7). Furthermore, to understand the manner in which electrostatic secondary‐sphere functionality affects CO_2_ reduction kinetics, we have plotted the partial catalytic currents corresponding to CO (*j*_CO_) and H_2_ (*j* ${}_{H{}_{2}}$ ) generation (Figure 4c). One can clearly observe that Zr‐BTB@Hemin‐TMA demonstrated remarkably higher j_CO_ in comparison to Zr‐BTB@Hemin, reaching up to 3‐times higher at −1.5 V vs. NHE. Nevertheless, both with and without TMA incorporation into the MOF, a rather similar j_H2_ was recorded, thus pointing to the fact that electrocatalytic rate of CO_2_ reduction to CO is greatly accelerated by the presence of the MOF‐tethered TMA (as opposed to a suppression of the competing H_2_ evolution reaction).

Next, we were interested in gaining further insights on the mechanisms governing the improved electrocatalytic CO_2_‐to‐CO performance of Zr‐BTB@Hemin‐TMA compared to Zr‐BTB@Hemin. First, Raman spectroscopy characterization of Zr‐BTB@Hemin and Zr‐BTB@Hemin‐TMA provided an indication of possible electronic communication between MOF‐installed Hemin and TMA ligands. Specifically, upon TMA‐tethering a shift of ν_2_ and ν_4_ symmetric pyrrole stretching bands toward higher frequencies (ν_2_ from 1367 to 1369 cm^−1^, and ν_4_ from 1562 to 1568 cm^−1^), indicating the conversion of low‐spin Fe^3+^‐Hemin into a high‐spin species (Figure S8).[^17d^, ^32^]

**Figure 5:**
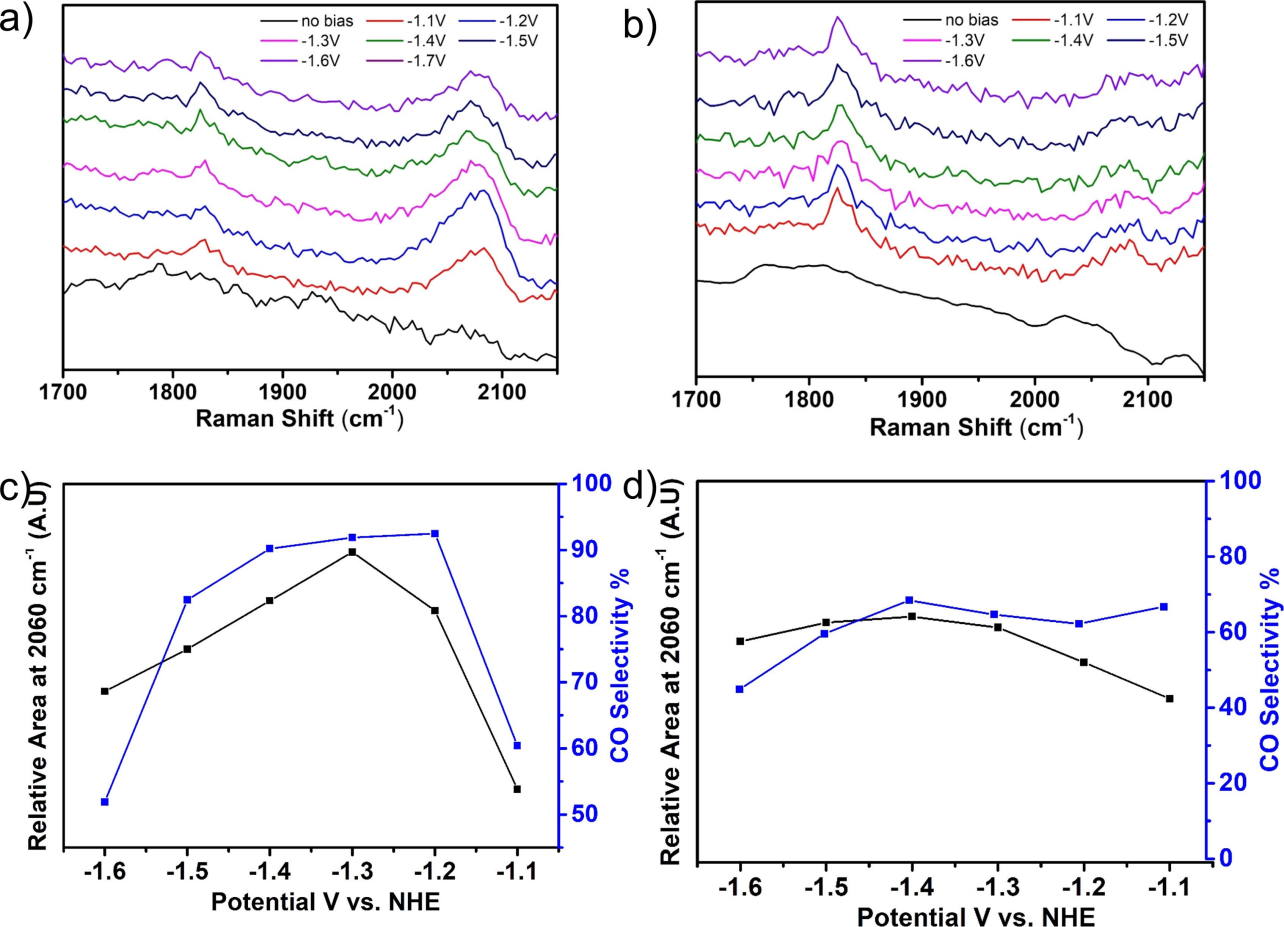
In situ Raman spectroscopy measurements conducted under working electrocatalytic conditions. a), b) Plots of Raman spectra recorded at different applied potentials (showing 2 types of Hemin‐bound CO intermediate species), for Zr‐BTB@Hemin‐TMA and Zr‐BTB@Hemin, respectively. c), d) Plots showing the correlation between the relative area of the 2060 cm^−1^ peak and the corresponding electrocatalytic CO selectivity, for Zr‐BTB@Hemin‐TMA and Zr‐BTB@Hemin, respectively.

Thereafter, we have conducted in situ Raman spectroscopy analysis under working electrocatalytic conditions, which allows the detection of reactive catalytic intermediates. In that manner, valuable information could be attained regarding the effect of TMA ligand on the MOF's electrocatalytic operation. In fact, in heme‐containing proteins it is well‐known that the IR stretching frequencies of Heme‐bound CO can serve as a sensitive gauge for electrostatic fields in close proximity of the CO binding site.^[33]^ Hence, we reasoned that by monitoring the stretching of Hemin‐CO intermediate, one would be able to probe TMA's effect on the system's electrocatalytic CO_2_ reduction properties. Generally, higher Raman shifts of CO stretching peaks is attributed to higher C−O bond order (and hence weaker Fe−C binding). As such, for both Zr‐BTB@Hemin‐TMA and Zr‐BTB@Hemin, in situ Raman measurements were performed at a set of applied potentials (−1.1 to −1.6 V vs. NHE) under CO_2_ reduction conditions (Figure 5). As seen in Figures 5a and b respectively, both samples exhibit two catalyst‐bound CO stretching peaks located at 1840 cm^−1^ and 2060 cm^−1^, albeit with opposite relative intensities. Namely, for Zr‐BTB@Hemin‐TMA the intensity of 2060 cm^−1^ peak is larger than that of the one at 1840 cm^−1^, and vice versa. Meaning, the MOF‐installed, cationic TMA ligand facilitates electrostatic stabilization of a more weakly‐bound CO intermediate, which can be swiftly released as a catalytic product (see illustrative Figure S9). Indeed, for all applied potentials, the relative intensity of the 2060 cm^−1^ peak relates to the CO selectivity trends of Zr‐BTB@Hemin‐TMA and Zr‐BTB@Hemin (Figures 5c and d).

**Figure 6:**
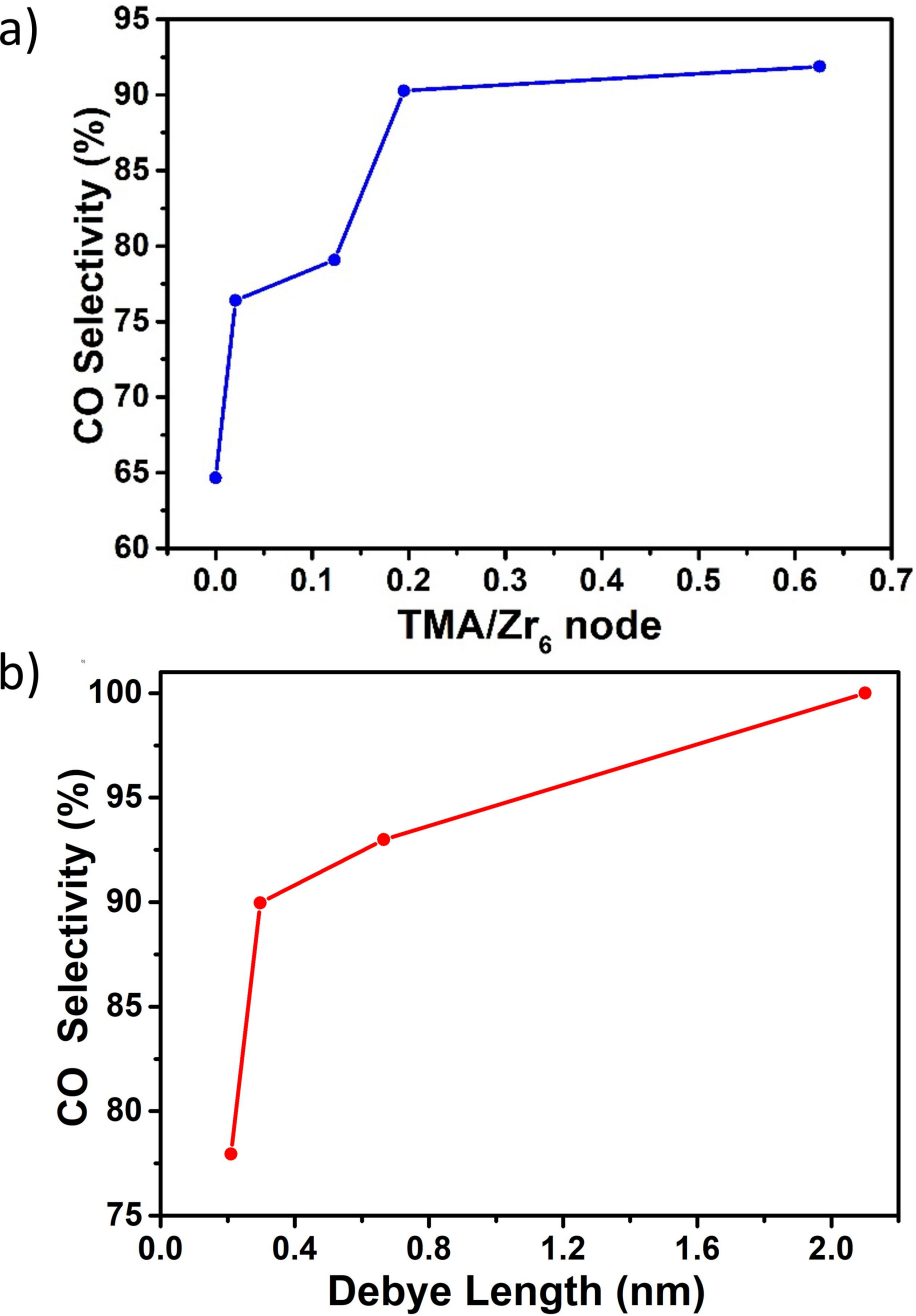
a) A plot of CO selectivity as a function of MOF‐installed TMA surface loading (TMA/Zr_6_‐oxo node). b) A plot of Zr‐BTB@Hemin‐TMA's (TMA/Zr_6_=0.63) CO selectivity as a function of electrolyte's Debye length. All measurements were conducted at an applied potential of −1.3 V vs. NHE.

At this point, we grasp that the installation of TMA ligand onto the MOF imposes a significant improvement in its electrochemical CO_2_ reduction performance by acting as electrostatic secondary‐sphere moieties. Yet, the extent in which one can control and regulate electrocatalysis using this concept remained unclear. Thus, to gain better understanding of the principles governing electrocatalytic operation in our MOF, we set to carry out the following experiments. First, we were interested to explore the manner in which TMA's surface concentration affect the MOF's electrocatalytic operation. To do so, we have compared the CO selectivity (measured at −1.3 V vs. NHE) of Zr‐BTB@Hemin‐TMA with varying TMA/Zr_6_ concentration ratio (see Figure S4a for experimental details). As shown in Figure 6a, Zr‐BTB@Hemin (no TMA) obtained CO selectivity of 65 %. By increasing the loading of TMA, a systematic improvement in catalytic selectivity was achieved, reaching up to 92 % (at TMA/Zr_6_ of 0.63). In other words, by increasing TMA surface loadings, the average Hemin‐to‐TMA distance is shortened, thus amplifying electrostatic secondary‐sphere interactions in our MOF system.

Secondly, it is well‐known that electrostatic potential in electrolytic solutions decay as a function of distance from a charged ion or surface, according to the electrolyte's Debye length, *κ* ^−1^:^[34]^

(1) $$\kappa^{-1}=\sqrt{\frac{\epsilon \epsilon_{0}RT}{2\times 10^{3}F^{2}I}}$$

Where ϵ is the solvent's dielectric constant, ϵ_0_ the permittivity of the vacuum, *I* is the electrolyte ionic strength, and *R*, *T* and *F* are the gas constant, temperature, and Faraday's constant, respectively. Hence, we have reasoned that by varying the electrolyte's ionic strength, one would be able to modulate *κ* ^−1^ and hence tune the extent of electrostatic interactions in the of Zr‐BTB@Hemin‐TMA system. To this end, we have compared the CO_2_‐to‐CO selectivity of BTB@Hemin‐TMA measured in different *κ* ^−1^, ranging from 2.1 nm to 0.2 nm (LiClO_4_ concentrations in the electrolyte 0.01–1 M). Indeed, as shown in Figure 6b, when high ionic strength is used (1 M LiClO_4_) TMA's charge is effectively screened within a short distance of 0.2 nm, thus lowering the probability of reaching a neighboring Hemin active site, diminishing CO selectivity down to 78 %. On the contrary, at low ionic strength (0.01 M LiClO_4_) the influence of the electrostatic field is felt even 2.1 nm away from the cationic TMA ligand. In this case, TMA's secondary‐sphere interactions are not restricted solely to adjacent Hemins (mounted on the same Zr_6_‐oxo node), but also to Hemins anchored on the next‐residing Zr_6_‐oxo node, resulting in an impressive 100 % CO_2_ conversion to CO. In addition, we have performed a control experiment, examining the effect of electrolyte ionic strength on the catalytic selectivity of Zr‐BTB@Hemin. As can clearly be seen in Figure S10, in contrast to Zr‐BTB@Hemin‐TMA, for low ionic strength (Debye length >0.6 nm) the CO selectivity of Zr‐BTB@Hemin remains constant (67 %). In other words, in the absence of TMA, no clear effect is seen when the electrostatic potential is felt for longer distances. We note that for lower Debye lengths, the CO selectivity drops. In this case, the higher electrolyte concentration resulted in increased catalytic current densities, thus causing a more rapid CO_2_ depletion near the catalyst surface, which accelerated H_2_ evolution. Consequently, these results further confirm that the incorporated TMA ligands affect the MOF's electrocatalytic properties via an electrostatic mechanism.

Finally, the stability of the Zr‐BTB@Hemin‐TMA electrocatalytic system was evaluated by a bulk‐electrolysis analysis conducted at −1.3 V vs. NHE. As shown in Figure S11a–c, over the entire period of measurement, Zr‐BTB@Hemin‐TMA sustained over 85 % of its initial CO selectivity. Furthermore, PXRD analysis and SEM images of Zr‐BTB@Hemin‐TMA before and after bulk‐electrolysis show the preservation of MOF crystal structure and morphology during electrocatalytic operation (Figure S11d–f). While no apparent decline in Hemin surface loading was detected, the density of MOF‐bound TMA decreased from 0.63 to 0.25 per Zr_6_‐oxo node (Figure S11g), thus explaining the slight drop in catalytic selectivity over time.

## Conclusion

In this work, we show that the electrocatalytic CO_2_ reduction performance of an Fe‐Porphyrin (Hemin)‐based MOF could be precisely tuned via electrostatic secondary‐sphere interactions. Specifically, we demonstrate that tethering of fixed cationic charge proximal to Zr‐BTB MOF‐installed Hemins, substantially enhance their electrochemical CO_2_‐to‐CO rate and selectivity. In situ Raman spectroscopy conducted under working electrochemical conditions, revealed that the improved electrocatalysis originated from electrostatic stabilization of a weakly‐bound CO intermediate, which accelerated its release as a catalytic product. Moreover, the degree of electrostatic field could be systematically manipulated by adjustment of either the density of charged ligands or electrolyte's ionic strength, resulting in practically 100 % CO_2_ conversion to CO. Notably, the presented concept provides a new perspective on MOFs capability of controlling the local chemical environment surrounding a catalytically active site. Hence, it should provide new means for molecular‐level modulation of heterogeneous electrocatalytic systems.

## Supporting Information

Additional experimental details, material characterizations, and electrochemical measurements are given via a link at the end of the document.

## Conflict of interest

The authors declare no conflict of interest.

1

## Supporting information

As a service to our authors and readers, this journal provides supporting information supplied by the authors. Such materials are peer reviewed and may be re‐organized for online delivery, but are not copy‐edited or typeset. Technical support issues arising from supporting information (other than missing files) should be addressed to the authors.

Supporting Information

## Data Availability

The data that support the findings of this study are available in the Supporting Information of this article.
