# Supplementary material for: Electrostatic Secondary‐Sphere Interactions That Facilitate Rapid and Selective Electrocatalytic CO2 Reduction in a Fe‐Porphyrin‐Based Metal–Organic Framework
Source: Angew Chem Int Ed Engl. 2022 Jun 28;61(32):e202206085. doi: 10.1002/anie.202206085 (PMC9401588; doi:10.1002/anie.202206085)
Supplement: Supplementary file 1 — Supporting Information [file ANIE-61-0-s001.pdf]

Angewandte Chemie
Eine Zeitschrift der Gesellschaft Deutscher Chemiker

Supporting Information

Electrostatic Secondary-Sphere Interactions That Facilitate Rapid and Selective Electrocatalytic CO_2 Reduction in a Fe-Porphyrin-Based Metal–Organic Framework

R. Shimoni, Z. Shi, S. Binyamin, Y. Yang, I. Liberman, R. Ifraemov, S. Mukhopadhyay, L. Zhang, I. Hod**

Table of Contents

Section	Page Number
**Table of Contents**	S2
**Experimental procedures**	S2
**Physical characterization**	S3-S4
**Result and discussions**	S5-S11

Experimental Procedures

The chemicals.

Zirconium (IV) chloride ($ZrCl_4 \geq 99.5\%$), 1,3,5-tris(4-carboxyphenyl)benzene-H_3BTB ($C_{27}H_{18}O_6 \geq 98\%$), lithium perchlorate ($LiClO_4, \geq 95\%$), 2-methyl imidazole ($C_6H_6N_2, 99\%$), Nafion perfluorinated solution (5 wt %), (3-carboxypropyl)trimethylammonium chloride (TMA-chloride, $C_7H_{16}NO_2Cl \geq 97\%$) and ferrocene ($Fe(C_5H_5)_2$, 98%) were purchased from Sigma-Aldrich. Hemin ($C_{34}H_{32}ClFeN_4O_4, \geq 98\%$) was purchased from Carl Roth. Dimethylformamide (DMF, C_3H_7NO) was purchased from Bio-Lab. Ethanol ($C_2H_6O, 99.7\%$) and acetonitrile $C_2H_3N, 99\%$ were purchased from J.T Baker. Carbon cloths (thickness:0.33mm, basic weight 120 g/m2, resistance <5 mΩcm2) were provided by Gochen ltd.

Synthesis of Zr-BTB nanosheet.

$ZrCl_4$ (100 mg), H_3BTB (100 mg), benzoic acid (6 g), water 5 ml, and DMF (30 mL) were charged in a 100ml cupped bottle. The mixture was sonicated until dissolved and then heated to 120 °C in the oven for 48 h. After cooling down to room temperature, the product was collected by centrifugation, and washed with DMF twice and acetone twice.

Post synthetic modification of Zr-BTB with Hemin.

80 mg (0.125 mmol) of Hemin was added to 10 ml of DMF containing 100 mg (0.0625 mmol) of Zr-BTB nanosheet powder. The suspension was heated at 70°C inside the oven overnight. The prepared Zr-BTB@Hemin was isolated from the solution by centrifugation and washed three times with DMF (or until the solution became colourless and three times with Ethanol. The product was dried at 70 °C overnight in the vacuum oven.

Carbon-cloth electrode preparation.

15 mg of Zr-BTB@Hemin were homogeneously dispersed in a solvent mixture of 0.75 ml ultra-pure water, 0.25 ml of isopropyl alcohol, and 70 µl of Nafion solution by ultra-sonication until a homogeneous ink was obtained. Then the carbon cloth was coated via the drop-casting method with small portions of this ink up to 330 µl so that loading of 5 mg/cm^2 (dry weight) could be maintained. The carbon-cloth coated with Zr-BTB@Hemin film was directly used as a working electrode, and for post syntactic modification using TMA.

Post synthetic modification of Zr-BTB@Hemin electrode with TMA.

5 mg (0.11 mmol) of TMA was dissolved in 5 ml of methanol in a screw-capped glass vial. Zr-BTB@Hemin coated carbon-cloth electrode was dipped inside the solution. The vial was heated to 50 ^{0}C for 12 hours. Then, the electrode was washed by immersion into 10 ml of fresh methanol at 50 ℃ for another 9 hours. Every 3 hours, the washing liquid was replaced with fresh methanol. Finally, the post-synthetically modified Zr-BTB@Hemin_TMA electrode was dried in a vacuum oven for 3 hours.

Physical characterization.

Physical characterization methods.

The as-prepared MOFs, Zr-BTB, Zr-BTB@Hemin, and Zr-BTB@Hemin_TMA crystalline structures were confirmed by X-ray diffraction using PANalytical's Empyrean multi-purpose diffractometer instrument and Cu-Kα (0.15405 nm) radiation.

Scanning electron microscope (SEM) images were recorded using Verios XHR 460L SEM instrument operating at 2kV accelerating voltage.
The zirconium and iron concentrations were determined by ICP-OES analysis. For ICP-OES, the Zr-BTB@Hemin was digested in concentrated nitric acid at 130 °C (overnight) . The diluted sample was analyzed using Spectro ARCOS ICP-OES, FHX22 multiView plasma (SOP, EOP) instrument.

Raman spectroscopic measurements were done in Horiba LabRam HR evolution micro-Raman system, equipped with a Synapse Open Electrode CCD detector air-cooled to -60 °C. The excitation source was a 532 nm laser, and it was focused with an x50 objective. The typical exposure time was 120-180 sec. The *in-situ* Raman spectra were measured on an XploRA confocal spectrometer (Jobin Yvon, Horiba Gr, France) with a charge coupled device (CCD) detector. Raman scattering was excited by an external-cavity diode laser (532 nm) coupled with a 50 **×** Olympus microscope objective. A CCD with 1024 × 256 pixels was used to collect the spectra in a resolution of 1.2 cm^{-1}, with 2 accumulations at 2s acquisition time.

Hydrogen Nuclear Magnetic Resonance (^1H-NMR) measurements were performed to evaluate the concentration of TMA in the post-synthetically modified Zr-BTB@Hemin_TMA. A calibration curve was prepared by dissolving different concentrations of TMA in D_2O with 1M NaOH. Then 1 mg of various sets of Zr-BTB_TMA samples, prepared using different concentration levels of TMA, was dissolved in 2M NaOH in D_2O and then diluted to 1M NaOH. A known amount of DMSO was added as an internal standard for each NMR tube.

TEM operated at 200 kV was used for high-resolution transmission electron microscopy (HRTEM) imaging and for the energy-filtered TEM (EFTEM). Energy-filtered TEM (EFTEM) experiments were performed using GATAN 894 US1000 camera and a Gatan image filter. Energy Dispersive X-ray Spectroscopy (EDS) and Electron Energy Loss Spectroscopy (EELS) analysis were performed to estimate the concentration ratio between Iron, Zirconium and Nitrogen. Sample preparation for HRTEM was done by sonication-assisted exfoliation of the samples from a solution prepared in isopropyl alcohol (IPA) (0.1 mg ml^{-1}) and sonicated for two hours. The solutions were centrifuged at 3000 rpm for 10 min. Then, ultra-thin carbon on lacey 400 mesh Cu (TED PELLA INC. 01824) was submerged in the suspension and lifted up to allow the IPA to evaporate. The coated grids were stored under vacuum overnight.

Atomic force microscopic (AFM) measurements were performed with the Instrument: Cypher-ES (Asylum Research/Oxford Instruments) Probe: HQ:NSC15/AL_BS (MikroMasch) Calibration: k = 27.4 N/m, DeflInvOLS = 46.8 nm/V Mode: AC-mode ("tapping mode"). Sample preparation was done by dispersing the MOF (Zr-BTB-TMA) in Ethanol. A small amount of the solution (2.5 µL) was placed on a freshly cleaved mica disc. After evaporation of the Ethanol, the mica substrate (glued onto an AFM specimen disc (Ted Pella, #16218)) was mounted on the AFM stage.

Electrochemical characterization.

Three-electrode two-compartment cell was used for electrochemical measurements. Ag wire and Pt foil (with an active area of 1 cm^2) were used as quasi-reference and counter electrodes, respectively. Zr-BTB@Hemin and Zr-BTB_TMA carbon-cloth electrodes were used as working electrodes (active area of 1 cm^2). Electrochemical measurements were performed with Bio-Logic VSP instrument. Before each measurement, the electrolyte solution (solution of lithium perchlorate and Trifluoroethanol (TFE) prepared in acetonitrile; concentration: 0.1M of lithium perchlorate and 0.1M of TFE) was purged with argon for 30 min. All cyclic voltammograms are reported versus NHE $\mathrm{E_{NHE} = E_{Ag} + 0.63 - E_{Fc\backslash Fc^+}(V\ vs\ Ag)}$). and were conducted at 100 mV/sec scan rate unless mentioned otherwise. To analyze and quantify the products formed from the catalytic CO_2 reduction reaction, chronoamperometric measurements were performed at different applied potentials between -1.1 V (vs. NHE) and -1.6 V (vs. NHE). For each measurement, a constant charge of 0.5 C was passed. Headspace samples were injected into a gas chromatograph (GC) to detect and quantify the amount of evolved CO and H_2.

Electrochemical measurements for Zr-BTB@Hemin-TMA with different TMA concentrations.

A set of electrodes with TMA loading was prepared by adding different concentrations of TMA (between 0.01 to 5mg/ml) during post-synthetic modification of Zr-BTB@Hemin electrodes. For conducting TMA loading NMR experiments, each electrode was washed with methanol and then dissolved in a 2M NaOH D_2O solution. Three-electrode two-compartment cell was used for electrochemical measurements. Ag wire and Pt foil (with an active area of 1 cm^2) were used as quasi-reference and counter electrodes. Zr-BTB@Hemin-TMA carbon-cloth electrodes were used as working electrodes (active area of 1 cm^2). Before each measurement the electrolyte solution (solution of lithium perchlorate and Trifluoroethanol (TFE) prepared in acetonitrile; concentration: 0.1M of $LiClO_4$ and 0.1M of TFE) was purged with argon for 30 min and then 30 min with CO_2. Chronopotentiometric measurements (at -1.3V vs. NHE, 0.5C) were performed for the five different working electrodes with different loading levels of TMA in the MOF. Gas chromatography (GC) was used for analyzing and quantifying the products.

Selectivity in different Debye lengths.

Using a similar electrochemical system to the one presented above, electrochemical measurements were performed where different concentrations of $LiClO_4$ were used, which affected the ionic strength of the electrolyte solution. The ionic strength also dictates the Debye length k^{-1}, which was calculated following the equation:

$$k^{-1} = \sqrt{\frac{\varepsilon\varepsilon_0 RT}{2 * 10^3 F^2 I}}$$

Where I is the ionic strength based on electrolyte addition, ε is the dielectric constant of the solvent (In MeCN ε = 38), ε_0 is the permittivity of the vacuum, and R, T and F are the gas constant, temperature, and Faraday's constant, respectively.
For each Debye length condition, chronopotentiometry measurements were performed at E= -1.3 V (vs. NHE), passing a charge of 0.5C. Gas chromatography (GC) was used to analyze and quantify the CO and H_2 products.

In-situ electrochemical Raman spectroscopic measurements.

In-situ electrochemical Raman spectroscopic measurements were performed in a custom-made three-electrode two-compartment cell. Ag wire and Pt foil (with an active area of 1 cm^2) were used as quasi-reference and counter electrodes. Zr-BTB@Hemin and Zr-BTB_TMA Cu foil were used as working electrodes (active area of 2 cm^2). The $LiClO_4$ concentration in the electrolyte solutions of different measurements was varied as 0.01, 0.1, 0.5 and 1M in acetonitrile, while a fixed concentration of 0.1M was maintained for TFE. The electrolyte solution was purged with argon and CO_2 consecutively, each for 30 min. Raman spectra were recorded while performing chronopotentiometry at different potentials between -1.1 V to -1.6 V (vs. NHE) to see changes in the intermediates during the catalytic reaction.

Stability test of Zr-BTB_TMA for electrocatalytic CO_2 reduction.

A custom-made Three-electrode two-compartment cell with a circulation of CO_2 gas was used for electrochemical stability measurements. Ag wire and Pt foil (with an active area of 1 cm^2) were used as quasi-reference and counter electrodes. Zr-BTB_TMA carbon-cloth electrode was used as working electrode (active area of 1 cm^2). Before each measurement, the electrolyte solution (0.1M $LiClO_4$ in acetonitrile and 0.1M TFE) was purged with Ar and CO_2 consecutively, each for 30 min. Chronopotentiometry measurement was performed at -1.3V vs. NHE for 3 hours. GC was used for analyzing and quantifying the products after charge pass of 0.5 C, 1 C and then after every one hour to compare and understand the product selectivity.

Results and Discussions.

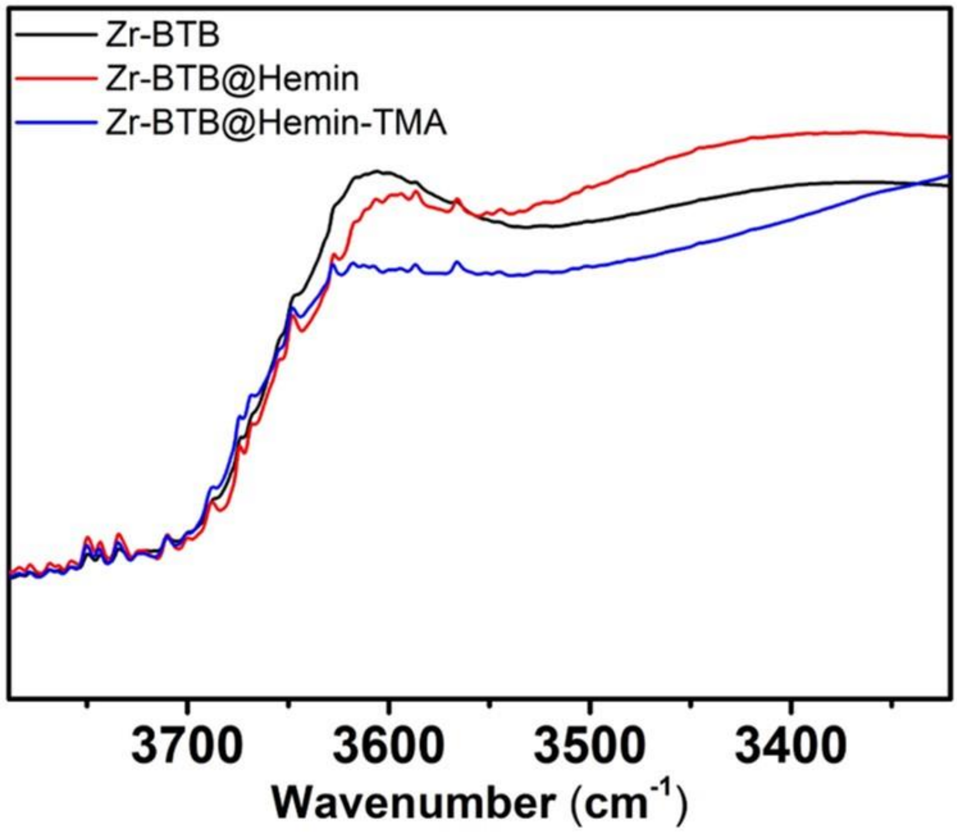

Figure S1. Fourier transformed infrared spectra of Zr-BTB, Zr-BTB@Hemin and Zr-BTB@Hemin-TMA.

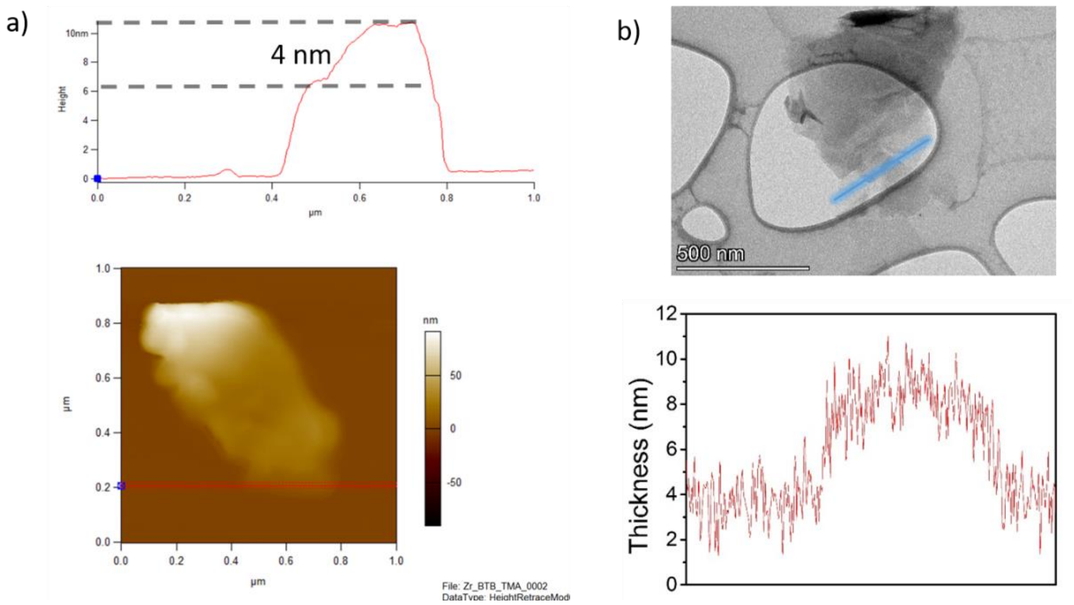

Figure S2. a) Thickness measurements using atomic force microscopy for Zr-BTB@Hemin-TMA. b) Thickness measurement of Zr-BTB@Hemin-TMA using Electron Energy Loss Spectroscopy.

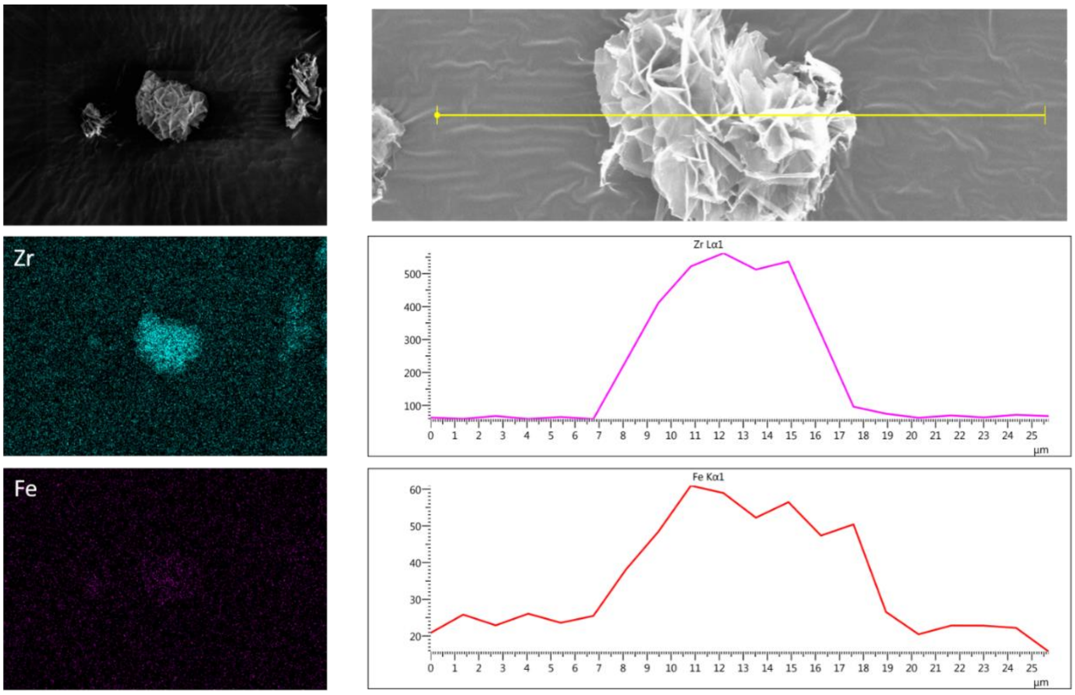

SEM		
Zr atomic%	Fe atomic%	Retio Fe/Zr_6
32.9	3.56	0.65

Figure S3. Energy Dispersive X-Ray Spectroscopy with elemental mapping and elemental line scan confirming the existence of Fe and Zr in Zr-BTB@Hemin-TMA.

a)

TMA mg/ml synthases solution	NMR integration	bind TMA concentration	TMA/Zr_6
0.01	0.015	0.008	0.02
0.1	0.087	0.048	0.12
0.5	0.138	0.076	0.19
**1**	**0.44**	**0.244**	**0.63**
5	0.44	0.244	0.63

b)

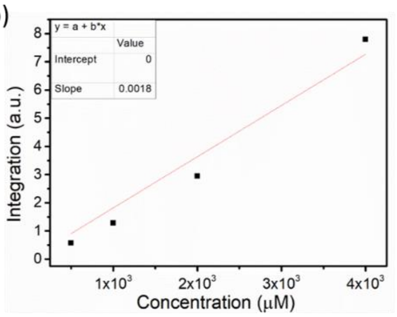

c)

	ICP-OES					NMR and ICP	
	Zr (mg/L)	Fe (mg/L)	Fe(umol)	Zr_6(umol)	Retio Fe/Zr_6	Ratio TMA/Zr_6	Ratio TMA/Fe
Zr-BTB@Hemin	12.6	1.1	0.31	0.35	0.88	-	-
Zr-BTB@Hemin-TMA	13.4	1.2	0.34	0.39	0.87	0.63	0.72
Zr-BTB@Hemin-TMA as powder	-	-	-	-	-	0.66	-

Figure S4. a) Summarizing table of the ^{1}H-NMR measurement of Zr-BTB@Hemin-TMA with different TMA concentrations. b) NMR calibration curve of TMA ligand. c) Summarizing table of ICP-OES and NMR results, showing the ratio of Fe/Zr_6 and TMA/Zr_6 for Zr-BTB@Hemin and Zr-BTB@Hemin-TMA.

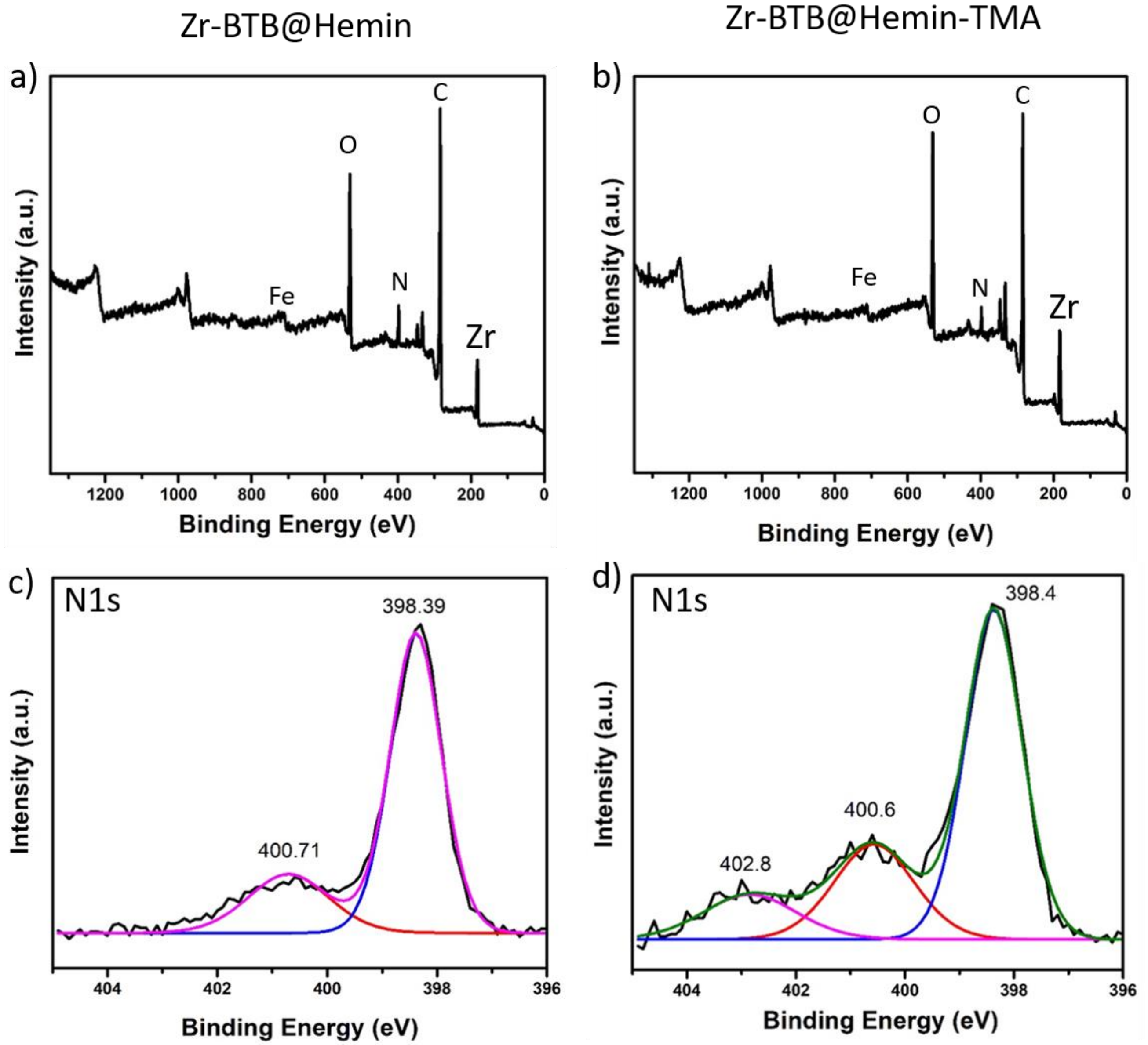

Figure S5. XPS mesurment showing the survey scans and the N1s analysis for Zr-BTB@Hemin (a,c) and Zr-BTB@Hemin-TMA (b,d).

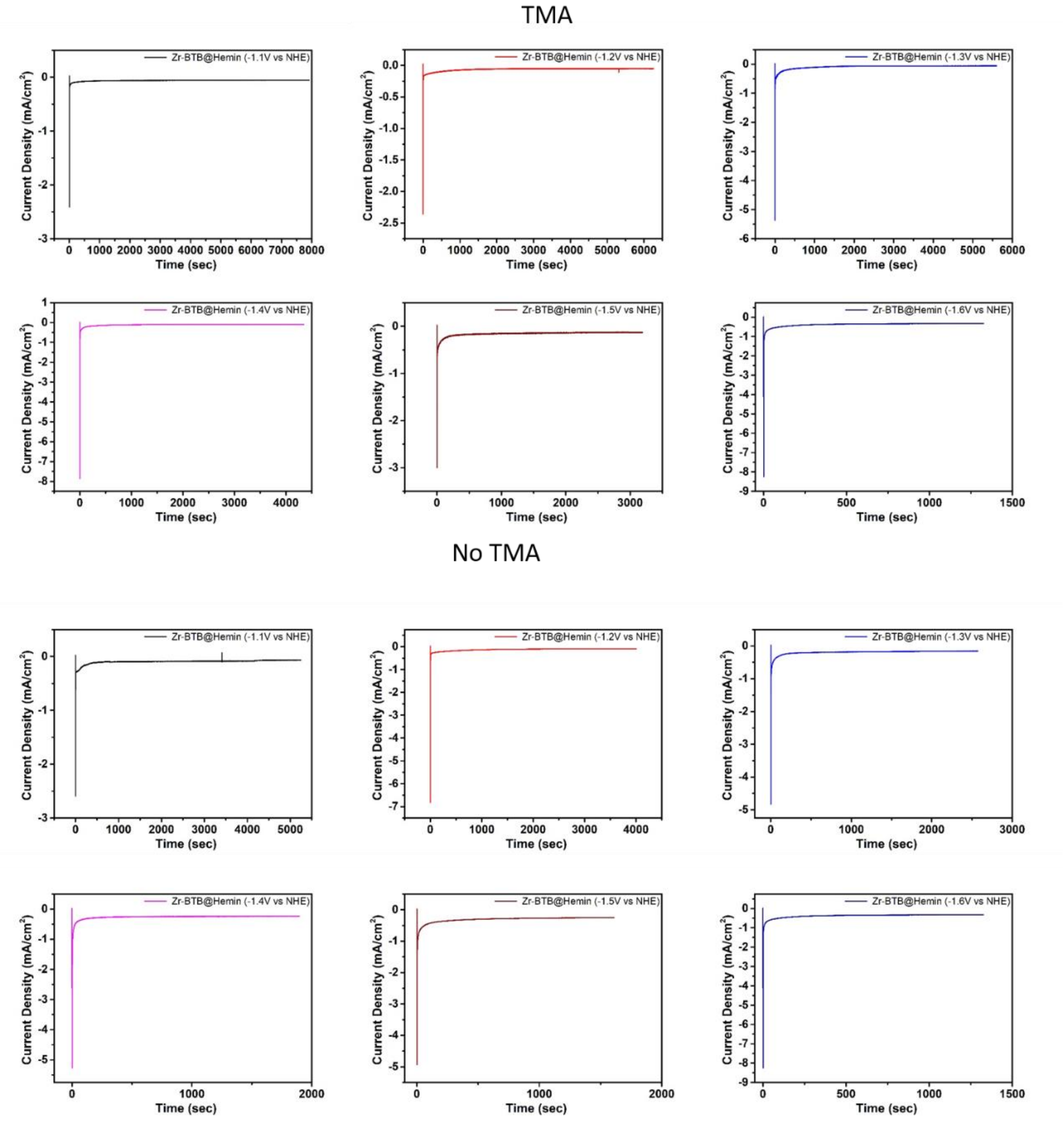

Figure S6. I-t curve of 0.5C chronoamperometric measurements for all voltages between -1.1 V to -1.6 V vs. NHE for both Zr-BTB@Hemin and Zr-BTB@Hemin-TMA.

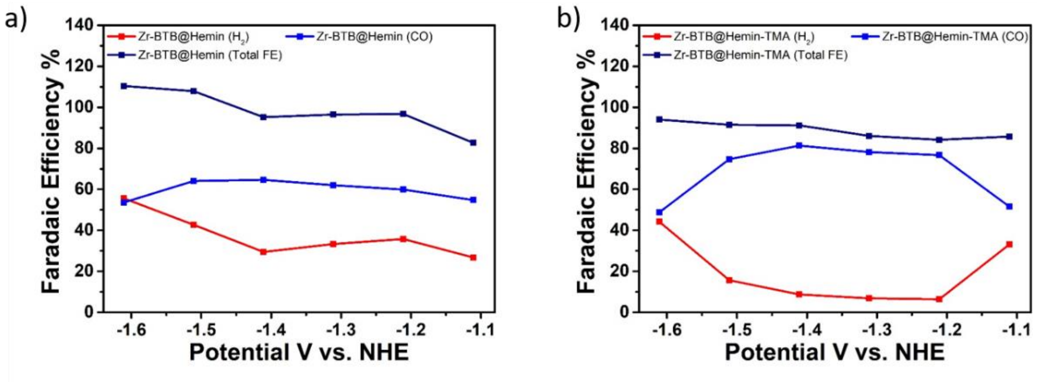

Figure S7. Presenting the Faradic efficiency of Zr-BTB@Hemin and Zr-BTB@Hemin-TMA in all chronoamperometric measurements of 0.5C

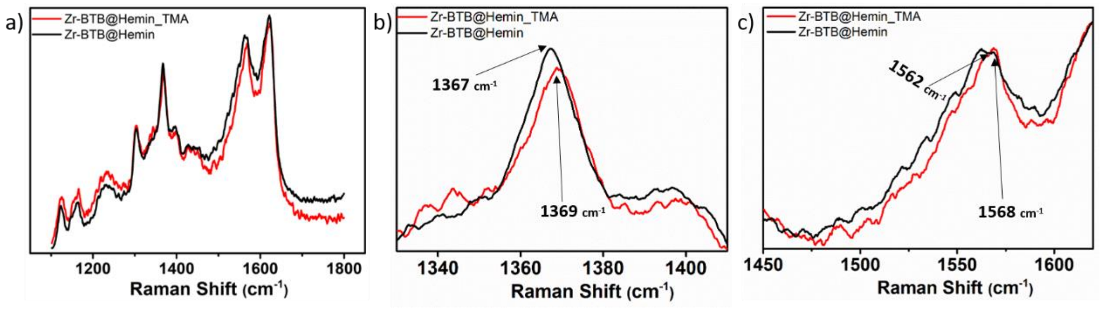

Figure S8. a) The Raman spectra of Zr-BTB@Hemin (black) and Zr-BTB@Hemin_TMA (red). b) Expansion of the region showing the v4 peak of the pyrrole half-ring stretching. c) Expansion of the region showing the v2 peak region of the pyrrole half-ring stretching.
The shift of the v_4 peak from 1367 cm^{-1} to a higher frequency of 1369 cm^{-1} indicates the coordination of TMA to the Hemin, changing its spin property toward its low spin form. This conclusion is further supported by the change in the ratio between the peaks at 1562 cm^{-1} (high spin) and 1568 cm^{-1} (low spin), v_2 stretching in the Zr-BTB@Hemin and Zr-BTB@Hemin_TMA

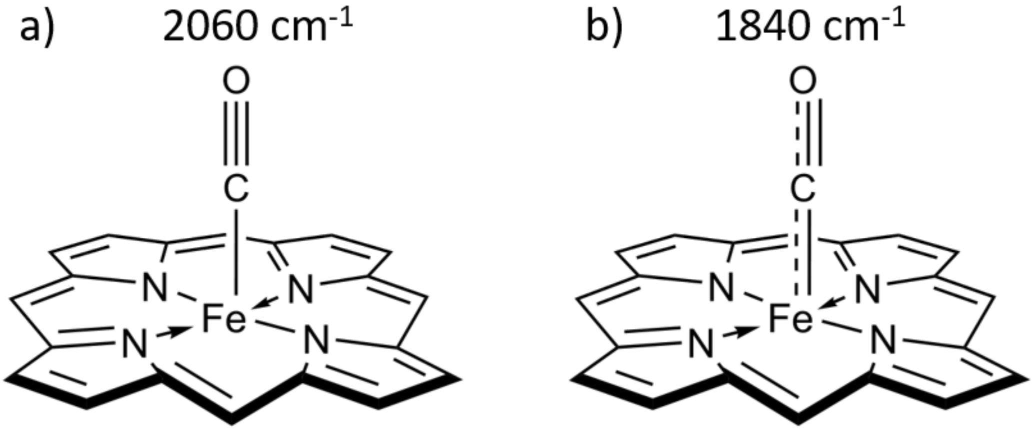

Figure S9. Schematic illustration showing the different C-O and Fe-C bond orders for the 2 Hemin-bound CO intermediates detected using in-situ Raman spectroscopy.

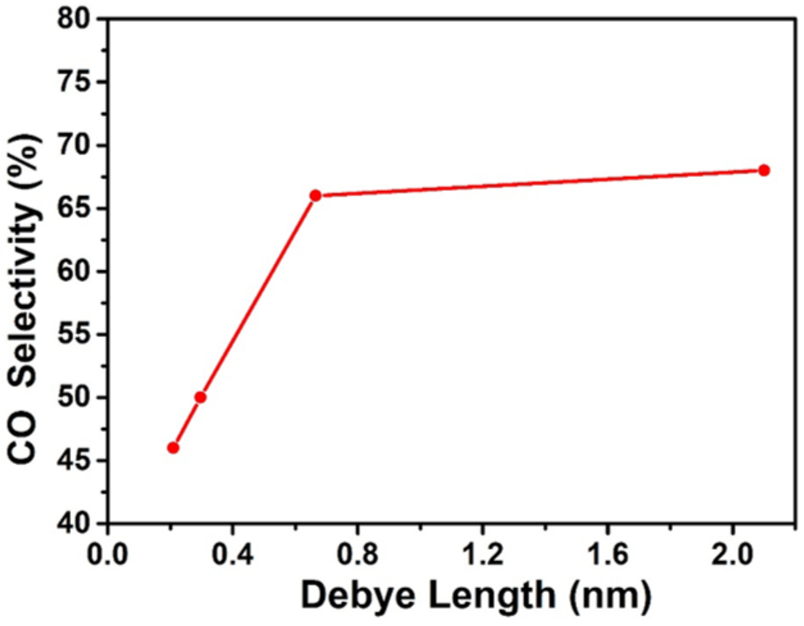

Figure S10. Mesurment of Zr-BTB@Hemin's CO selactvity as a function of electrolyte's Debye length.

a)

time (s)	Charge (C)	Selectivity to H_2 %	Selectivity to CO %
500	0.5	5.43	94.57
1150	1	13.56	86.44
3600	2.1	16.65	83.35
7200	2.97	18.60	81.40
10800	3.6	19.03	80.97

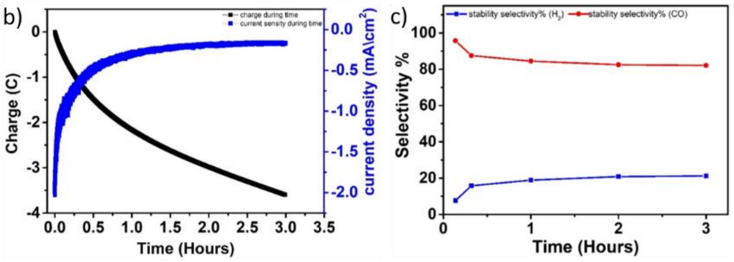

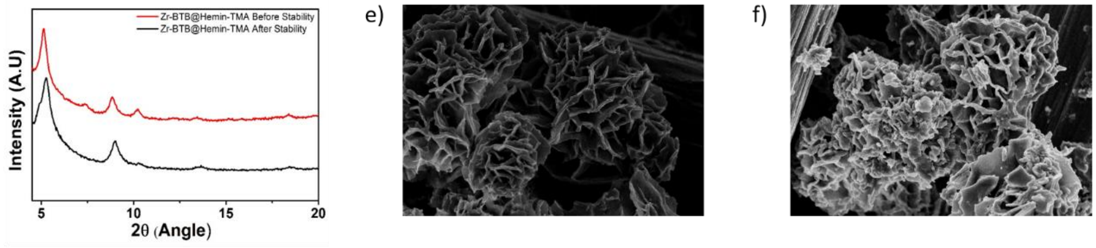

g)

	ICP-OES					NMR and ICP
	Zr (mg/L)	Fe (mg/L)	Fe(umol)	Zr_6(umol)	**Ratio Fe/Zr_6**	**Ratio TMA/Zr_6**
Before stability	13.4	1.2	0.34	0.39	**0.87**	**0.63**
After stability	1.2	0.101	0.31	0.35	**0.83**	**0.25**

Figure S11. a) table summarizing the 3 hour stability mesurment and the selectivity to CO and H2 b) presenting Chronoamperometry measurement of 3 hour c) graphic representation of selectivity to CO and H2 d) PXRD of Zr-BTB@Hemin_TMA before and after three-hour stability measurement. e,f) SEM images of Zr-BTB@Hemin_TMA before and after three-hour stability measurement respectively g) A table presenting the loading of Hemin (Fe) and TMA per Zr6-oxo node.

Author Contributions

Idan Hod and Liwu Zhang supervised this project. Ran Shimoni carried out the project. Ran Shimoni conducted experiments and analyzed data. Shahar Binyamin performed the XRD measurements. Itamar Liberman performed the ICP-OES measurements and SEM measurements. Raya Ifreamov performed the Raman measurements. Zhuocheng Shi and Yang Yang performed the Electrochimical In-Situ Raman measurements. Idan Hod and Ran Shimoni wrote the paper.
